# Tuning Cu-Content La_1−x_Sr_x_Ni_1−y_Cu_y_O_3−δ_ with Strontium Doping as Cobalt-Free Cathode Materials for High-Performance Anode-Supported IT-SOFCs

**DOI:** 10.3390/ma15248737

**Published:** 2022-12-07

**Authors:** Jakub Lach, Kun Zheng, Ryszard Kluczowski, Anna Niemczyk, Hailei Zhao, Min Chen

**Affiliations:** 1 Department of Hydrogen Energy, Faculty of Energy and Fuels, AGH University of Science and Technology, al. A. Mickiewicza 30, 30-059 Krakow, Poland; 2 AGH Centre of Energy, AGH University of Science and Technology, ul. Czarnowiejska 36, 30-054 Krakow, Poland; 3 Ceramic Department CEREL, Institute of Power Engineering, Techniczna 1, 36-040 Boguchwala, Poland; 4 Institute of Power Engineering, Mory 8, 01-330 Warsaw, Poland; 5 Center for Hydrogen Technologies (CTH2), Institute of Power Engineering, Augustowka 36, 02-981 Warsaw, Poland; 6 School of Materials Science and Engineering, University of Science and Technology Beijing, Beijing 100083, China; 7 Beijing Key Lab. of New Energy Materials and Technology, Beijing 100083, China; 8 School of Materials Science and Energy Engineering, Foshan University, Foshan 528000, China

**Keywords:** cathode materials, Cu-rich perovskites, Sr doping in (LaSr)(NiCu)O_3_, intermediate-temperature solid oxide fuel cells, anode-supported SOFCs

## Abstract

Cu-content La_1−x_Sr_x_Ni_1−y_Cu_y_O_3−δ_ perovskites with A-site strontium doping have been tuned as cobalt-free cathode materials for high-performance anode-supported SOFCs, working at an intermediate-temperature range. All obtained oxides belong to the *R*-3*c* trigonal system, and phase transitions from the *R*-3*c* space group to a *Pm*-3*m* simple perovskite have been observed by HT-XRD studies. The substitution of lanthanum with strontium lowers the phase transition temperature, while increasing the thermal expansion coefficient (TEC) and oxygen non-stoichiometry δ of the studied materials. The thermal expansion is anisotropic, and TEC values are similar to commonly used solid electrolytes (e.g., 14.1 × 10^−6^ K^−1^ for La_0.95_Sr_0.05_Ni_0.5_Cu_0.5_O_3−δ_). The oxygen content of investigated compounds has been determined as a function of temperature. All studied materials are chemically compatible with GDC-10 but react with LSGM and 8YSZ electrolytes. The anode-supported SOFC with a La_0.95_Sr_0.05_Ni_0.5_Cu_0.5_O_3−δ_ cathode presents an excellent power density of 445 mW·cm^−2^ at 650 °C in humidified H_2_. The results indicate that La_1−x_Sr_x_Ni_1−y_Cu_y_O_3−δ_ perovskites with strontium doping at the A-site can be qualified as promising cathode candidates for anode-supported SOFCs, yielding promising electrochemical performance in the intermediate-temperature range.

## 1. Introduction

Various types of energy storage and conversion technology are under development to balance the mismatch of supply and demand for energy sources, including wind and solar renewables, which are considered to be a form of intermittent power and connected with numerous aspects, such as weather variations and geographic location. The solid oxide fuel cell (SOFC) is one of the most favorable energy conversion and storage devices, which can be scaled up for decentralized energy applications [1,2,3,4]. SOFCs possess the capability to produce electricity and heat using the fuel and to store surplus electricity when demand is low in the fuel within electrolysis mode (the reversed operation of SOFC). Good power yields (exceeding 1000 mW·cm^−2^) of SOFCs are usually observed at a rather high temperature range (above 800 °C) [5]. The high working temperature of SOFCs leads to considerably high operational costs, and it also limits the choice of device materials, making SOFCs still unmarketable. Therefore, the commercial application of SOFCs requires a lowering of the operation temperature to an intermediate range (500–750 °C), while still maintaining high cell power density [6,7]. To bring down the working temperature of SOFCs, electrodes with highly electrocatalytic activity and stability are required to enable a reasonable power output. For intermediate-temperature solid oxide fuel cells (IT-SOFCs), the electrochemical performance deterioration of the cathode at reduced temperatures has a huge impact on output power. An effectively working cathode with excellent efficiency in oxygen reduction and evolution reactions at an intermediate-temperature range is a requisite to providing the stable and high performance of IT-SOFCs [7,8,9].

The perovskite (ABO_3−δ_) or perovskite-related structured oxide is one group of the most interesting and comprehensively studied cathode material candidates for IT-SOFCs, presenting great potential in chemical composition modifications, yielding the design and gain of desired physicochemical (including mixed ionic–electronic transport properties) and electrochemical properties [7,10]. Cobalt-based perovskites, including La_1−x_Sr_x_Co_1−y_Fe_y_O_3−δ_ [11,12,13] and Ba_1−x_Sr_x_Co_1−y_Fe_y_O_3−δ_ compounds [9,13], were systematically investigated as cathode materials for IT-SOFCs, presenting promising mixed ionic–electronic conductivity and excellent electrocatalytic reactivity for oxygen reduction reactions [14,15]. In addition, double perovskites with a formula of Ln_2−x_(Ba,Sr)_x_Co_2−y_M_y_O_5+δ_ (Ln: lanthanides M: 3*d* metals) [16,17,18,19] present very fast oxygen ionic transport, related to the layered structure, contributing to a favorable performance in IT-SOFCs. However, the shortcomings of cobalt-containing compounds related to the very high thermal expansion coefficient [20,21,22], negative environmental impact, and high price of cobalt [23,24] significantly limit their commercial applications. Therefore, the development of cobalt-free alternatives with high performance is of importance [25,26]. Cu-content materials featuring favorable physicochemical properties belong to the group of promising alternative cathode materials for SOFCs [25]. For example, La_4_BaCu_5_O_13±δ_, featuring a low cathodic polarization value of 0.03 Ω·cm^2^ at 900 °C, was proposed as a novel cathode for SOFCs, enabling the achievement of a favorable power yield exceeding 1000 mW·cm^−2^ at 900 °C [27]. The triple perovskite La_1.5_Ba_1.5_Cu_3_O_7±δ_ was investigated as a Co-free cathode candidate for SOFCs, exhibiting a very low polarization value of 0.019 Ω·cm^2^ and a relatively high performance of 458 mW·cm^−2^ at 750 °C [28]. The Ln(Ba,Sr)Cu_2_O_5+δ_ (Ln: Nd and Sm)-layered double perovskites were also studied as cathode candidates for IT-SOFCs, presenting relatively low thermal expansion coefficients and good electrochemical properties [29,30,31]. Ln_2_CuO_4+δ_-type (Ln: lanthanides) Ruddlesden–Popper oxides with the presence of interstitial oxygen favoring ionic transport were systematically explored as new cathodes for SOFCs [32,33,34,35].

The simple perovskite LaCuO_3_ is one of the well-studied Cu-content oxides with a superior high conductivity (10^6^ S·cm^−1^) [36]. However, the stoichiometric LaCuO_3_ perovskite can be hardly obtained and suffers with stability issues in air [37,38]. The cation-doping strategy should be applied to stabilize the perovskite structure. It has been noted that the LaCo_0.4_Ni_0.4_Cu_0.2_O_3−δ_ simple perovskite possesses very high electrical conductivity (1480 S·cm^−1^ at 500 °C), yielding a good peak power output at 700 °C (535 mW·cm^−2^) [39]. For Cu- and Ni-containing LaNi_0.5_Cu_0.5_O_3−δ_ compounds, a desirable low cathodic polarization of 0.056 Ω·cm^2^ was achieved at 800 °C, and a relatively high power output of 870 mW·cm^−2^ was recorded at 900 °C [40]. The generation of oxygen vacancies can be particularly advantageous for cathode materials, favoring an increase in the ionic conductivity component [41]. The beneficial effect of strontium doping in the La_2−x_Sr_x_NiO_4+δ_ system was reported to enhance the structure stability of La_2_NiO_4_ by increasing the bond length of La(Sr)–O [42]. The substitution of La with Sr in La_2−x_Sr_x_NiO_4+δ_ materials is favorable, contributing to the reduction in cathodic polarization and the increase in SOFC power density [43]. The valuable outcome of the Sr dopant was also confirmed by the reduction in oxygen vacancy formation energy in perovskite oxides La_1−x_Sr_x_MO_3−δ_ (M = Fe, Mn) [44]. Therefore, in this work, Cu-content La_1−x_Sr_x_Ni_1−y_Cu_y_O_3−δ_ oxides with strontium doping at the A-site were evaluated as very promising cobalt-free cathode material candidates for IT-SOFCs. The introduction of strontium at the A-site should result in an increase in oxygen non-stoichiometry δ in the proposed compounds. Physicochemical properties regarding crystal structure, phase transition, thermal expansion properties, oxygen content change as a function of temperature, chemical stability, and the compatibility of studied materials with commonly used solid electrolytes, as well as the electrochemical performance, were systematically investigated.

## 2. Materials and Methods

Soft chemistry methods were applied to synthesize the La_1−x_Sr_x_Ni_1−y_Cu_y_O_3−δ_ oxides. Stoichiometric amounts of La_2_O_3_, SrCO_3_, Ni(NO_3_)_2_·6H_2_O, and Cu(NO_3_)_2_·6H_2_O (all with purity ≥99.9%) were respectively dissolved in a HNO_3_ solution. Then, citric acid and ethylenediaminetetraacetic acid (as the complexing agent) were added during stirring at a molar ratio of 1:1 and 1.5:1, respectively, in relation to the total amount of all cations, and ammonia was added to neutralize the solutions to a pH value of 7. The obtained homogeneous solutions were slowly heated in quartz containers to around 400 °C. During the heating process, water evaporation, the decomposition of excessive ammonia nitrates and the oxidation of residual carbon occurred. The obtained precursors were well grounded and fired in air at 800 °C for 12 h. The La_1−x_Sr_x_Ni_0.75_Cu_0.25_O_3−δ_ (x = 0 and 0.05) and LaNi_0.5_Cu_0.5_O_3−δ_ compounds were successfully synthesized in air at 800 °C for 12 h. For the La_0.9_Sr_0.1_Ni_0.75_Cu_0.25_O_3−δ_ and La_0.95_Sr_0.05_Ni_0.5_Cu_0.5_O_3−δ_ oxides, additional heating, regrinding, and sintering at 800 °C for 12 h in pure oxygen were conducted to obtain single-phase materials. However, the synthesis of materials with a further increase in strontium doping did not succeed, despite trying additional heating, regrinding, and sintering at different temperatures (800–1000 °C) and atmospheres (air, oxygen and argon).

The crystal structure at room temperature (RT) of the obtained compounds was investigated by XRD studies using a Panalytical Empyrean diffractometer in the 10–110 deg range with CuKα radiation. High-temperature XRD (HT-XRD) studies were performed on a Panalytical Empyrean apparatus equipped with an Anton Paar HTK 1200N (Graz, Austria) oven chamber. The refinement of the collected XRD data was performed using the Rietveld method with a GSAS/EXPGUI-II set of software [45,46]. Particle size analysis of the powders of La_1−x_Sr_x_Ni_0.75_Cu_0.25_O_3−δ_ (x = 0, 0.05 and 0.1) and La_1−x_Sr_x_Ni_0.5_Cu_0.5_O_3−δ_ (x = 0 and 0.05) was performed using the Mastersizer 3000 laser-diffraction particle-size analyzer (Malvern Panalytical, Malvern, UK). Scanning electron microscopy (SEM) measurements were performed using ThermoFisher Scientific Phenom XL Desktop SEM apparatus on the powders obtained (Waltham, MA, USA). Thermal expansion studies of sinters in air up to 800 °C were carried out on a Linseis L75 Platinum Series dilatometer (Selb, Germany). Titration measurements were performed to determine the oxygen content in the studied materials using the EM40-BNC Mettler Toledo titrator with a platinum electrode (Mettler-Toledo, Poland). The oxygen content of the investigated compounds was calculated using the average values from three titration measurements. Thermogravimetric (TG) measurements were performed on TA Instruments Q5000IR (New Castle, DE, USA) apparatus from RT to 800 °C, with a heating rate of 2°·min^−1^, and the buoyancy effect was taken into account. The chemical stability and compatibility studies of the La_1−x_Sr_x_Ni_0.75_Cu_0.25_O_3−δ_ (x = 0, 0.05 and 0.1) and La_1−x_Sr_x_Ni_0.5_Cu_0.5_O_3−δ_ (x = 0 and 0.05) oxides towards typical solid electrolytes CGO10 (Ce_0.9_Gd_0.1_O_1.95_), LSGM (La_0.8_Sr_0.2_Ga_0.8_Mg_0.2_O_3−d_), and 8YSZ (8 mol% yttria stabilized zirconia) were studied by analyzing the collected XRD data for the respective compound and solid electrolyte mixtures (with a ratio of 50:50 wt.%), which were fired in air at 800 °C for 100 h.

As the anode-supported SOFC design considerably decreases the cell’s ohmic resistance and maximizes the power output [47], in this work, anode-supported IT-SOFCs were fabricated with the considered cathode material. The anode-supported half-cells with Ni-8YSZ | 8YSZ | CGO10 configuration were provided by the Ceramic Department CEREL, Institute of Power Engineering, Poland. The anode functional layer (around 7 µm) was deposited on the anode substrate of 1000 µm, and the 8YSZ electrolyte (~6 µm) with a CGO10 buffer (~6 µm) was applied. The details of the standard fabrication procedures of the anode-supported half-cells at the Institute of Power Engineering can be found in [48,49]. Cathode paste was prepared by the well mixing of grinded cathode material powder with an appropriate amount of a texanol-based binder, and the cathode layer (with a thickness of ~30 µm) was fired at 800 °C for 2 h in air. The area of the cathode in the constructed cells was approx. 0.25 cm^2^. Pt wires and Ag mesh were used as current collectors in tested cells. Cells were fueled by wet (ca. 3 vol% H_2_O) H_2_ with a gas flow of 40 cm^3^ min^−1^ and air flow for the cathode. SOFC performance was characterized using the Solartron SI 1287 interface and Solartron 1252A analyzer. Impedance spectroscopy studies were conducted under open-circuit conditions with a 25 mV amplitude in a 0.1–300 kHz range. The electrochemical impedance spectroscopy data were fitted with a L-R_ohm_-(RQ)_HF_-(RQ)_LF_ equivalent circuit, where L represents the inductance, R_ohm_—ohmic represents the resistance, and RQ is the resistance and constant phase elements, which can be related to processes occurring at high frequencies (HFs) and low frequencies (LFs) [50].

## 3. Results and Discussion

### 3.1. Crystal Structure Properties and Microstructure

**Figure 1:**
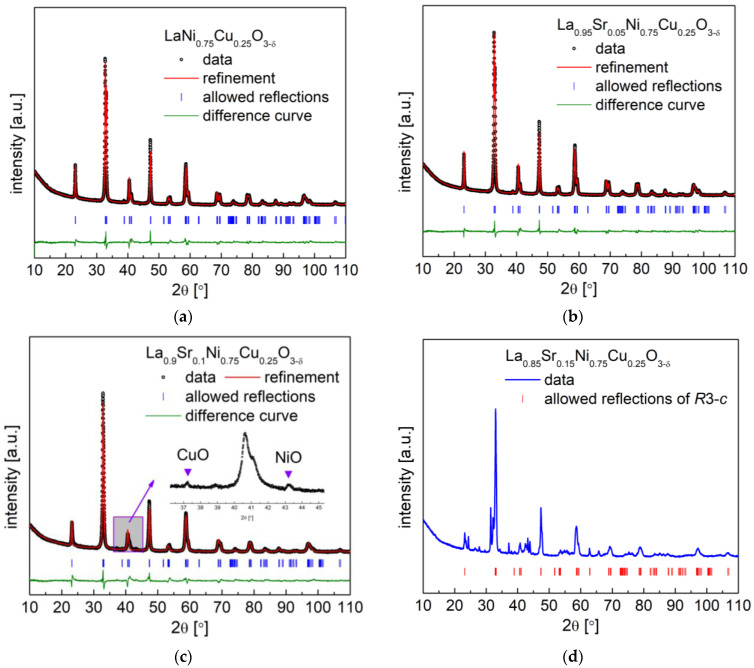
XRD patterns with Rietveld refinement recorded for La_1−x_Sr_x_Ni_0.75_Cu_0.25_O_3−δ_ oxides with (**a**) x = 0; (**b**) x = 0.05; (**c**) x = 0.1; (**d**) XRD patterns of La_0.85_Sr_0.15_Ni_0.75_Cu_0.25_O_3−δ_ oxides.

As reported in our previous work [40], high Cu-content LaNi_1−y_Cu_y_O_3−δ_ perovskites present attractive physicochemical and electrochemical properties as air electrode materials for SOFCs, especially LaNi_0.5_Cu_0.5_O_3−δ_ cathode material. The substitution of La with Sr at the A-site of La_1−x_Sr_x_Ni_1−y_Cu_y_O_3−δ_ perovskites contributes to an increase in oxygen vacancies in the compounds, thus enhancing ionic conductivity. As shown in Figure 1, the above-described soft chemistry synthesis method yielded La_1−x_Sr_x_Ni_0.75_Cu_0.25_O_3−δ_ (x = 0 and 0.05) single-phase compounds without the presence of any impurities. Meanwhile, for the La_0.9_Sr_0.1_Ni_0.75_Cu_0.25_O_3−δ_ oxide, very minor CuO and NiO secondary phases were observed, and a further introduction of strontium at the A-site led to the presence of a large number of impurities (see Figure 1d). Therefore, the maximum doping level of strontium in La_1−x_Sr_x_Ni_0.75_Cu_0.25_O_3−δ_ is limited to x = 0.1. The crystal structure of La_1−x_Sr_x_Ni_0.75_Cu_0.25_O_3−δ_ (x = 0, 0.05 and 0.1) can be refined using a rhombohedral structure with the *R*-3*c* space group, typical for the LaNiO_3_ [51], LaCuO_3_ [37], and Cu-containing LaNi_0.75_Cu_0.25_O_3−δ_ [40] perovskites. Rietveld refinement results for La_1−x_Sr_x_Ni_0.75_Cu_0.25_O_3−δ_ (x = 0, 0.05 and 0.1), including unit cell parameters and volume, are gathered in Table 1. As can be derived from the results, the increase in Sr content at the La-site causes a decrease in the unit cell volume of La_1−x_Sr_x_Ni_0.75_Cu_0.25_O_3−δ_ (x = 0, 0.05 and 0.1) (Table 1). This is related to the fact that an increase in Sr content causes an increase in the concentration of oxygen vacancies [52] and the average oxidation states of B-site cations, which were confirmed by the following TG measurements and titration analysis. In addition, B-site cations with high oxidation states strengthen the B-O bond in the BO_6_ structure block, thus decreasing the unit cell volume of the perovskite. The observed decrease in density with the increase in Sr doping for La_1−x_Sr_x_Ni_0.75_Cu_0.25_O_3−δ_ (x = 0, 0.05 and 0.1) oxides was due to the substitution of heavy lanthanum with light strontium.

**Figure 2:**
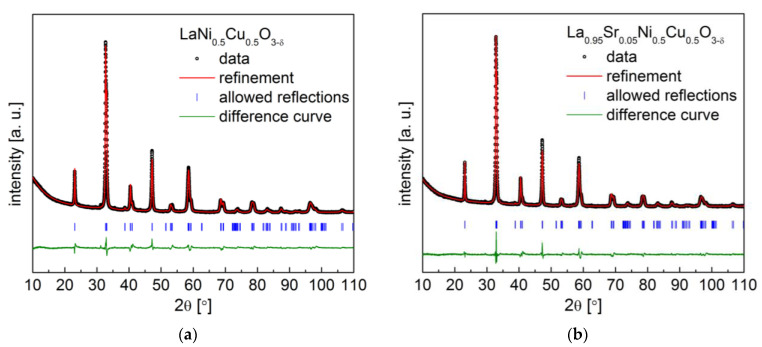
XRD patterns with Rietveld refinement recorded for La_1−x_Sr_x_Ni_0.5_Cu_0.5_O_3−δ_ oxides with (**a**) x = 0; (**b**) x = 0.05.

XRD data, together with Rietveld refinement for the La_1−x_Sr_x_Ni_0.5_Cu_0.5_O_3−δ_ (x = 0 and 0.05) oxides, are presented in Figure 2, and the refined data are shown in Table 1. However, further strontium doping in La_1−x_Sr_x_Ni_0.5_Cu_0.5_O_3−δ_ did not succeed. Sr doping did not change the crystal structure of the studied materials. LaNi_0.5_Cu_0.5_O_3−δ_ and La_0.95_Sr_0.05_Ni_0.5_Cu_0.5_O_3−δ_ compounds possess the same crystal structure as the *R*-3*c* space group. In the La_1−x_Sr_x_Ni_0.5_Cu_0.5_O_3−δ_ (x = 0 and 0.05) oxides, the presence of strontium at the A-site led to a reduction in the unit cell volume and density, which was also observed in the series of La_1−x_Sr_x_Ni_0.75_Cu_0.25_O_3−δ_ (x = 0, 0.05 and 0.1) perovskites.

**Figure 3:**
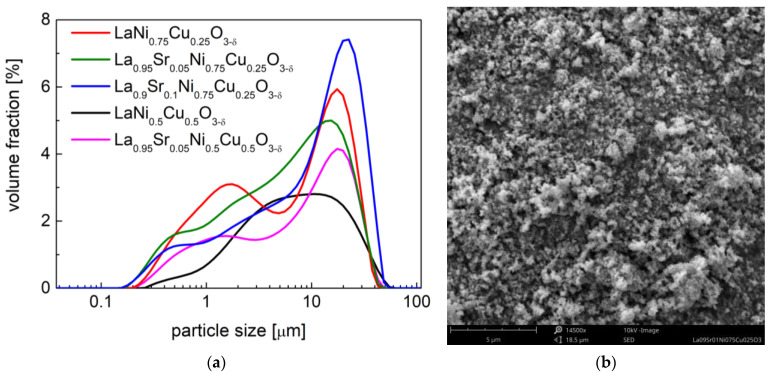
(**a**) Particle size analysis results of La_1−x_Sr_x_Ni_1−y_Cu_y_O_3−δ_ powders; (**b**) exemplary scanning electron micrograph of La_0.9_Sr_0.1_Ni_0.75_Cu_0.25_O_3−δ_ perovskite.

As presented in Figure 3, the microstructure studies of the La_1−x_Sr_x_Ni_0.75_Cu_0.25_O_3−δ_ (x = 0, 0.05 and 0.1) samples and La_1−x_Sr_x_Ni_0.5_Cu_0.5_O_3−δ_ (x = 0 and 0.05) powders show the presence of both small particles (≤1 µm) and larger aggregates (around 20 µm). The grain size of the studied materials is smaller than 1 µm, and all materials tend to form agglomerates, which results from that the fact that forming agglomerates can reduce the large specific surface area of the small powders. For the studied powders, no correlation was found between the content of strontium and the particle size distribution of all the investigated materials.

**Figure 4:**
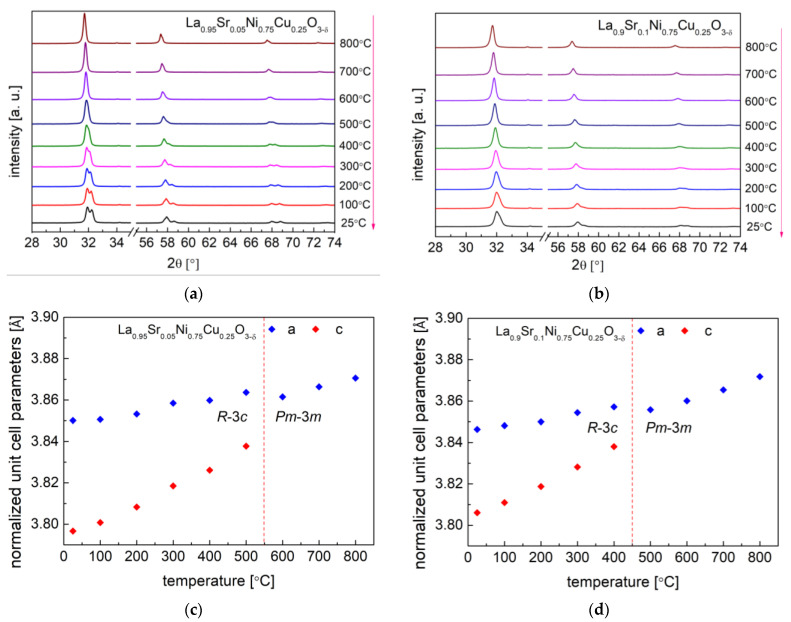
HT-XRD diffractograms recorded during the cooling from 800 °C to 25 °C in air for: (**a**) La_0.95_Sr_0.05_Ni_0.75_Cu_0.25_O_3−δ_ and (**b**) La_0.9_Sr_0.1_Ni_0.75_Cu_0.25_O_3−δ_; Normalized unit cell parameters dependence on temperature for (**c**) La_0.95_Sr_0.05_Ni_0.75_Cu_0.25_O_3−δ_ and (**d**) La_0.9_Sr_0.1_Ni_0.75_Cu_0.25_O_3−δ_.

**Figure 5:**
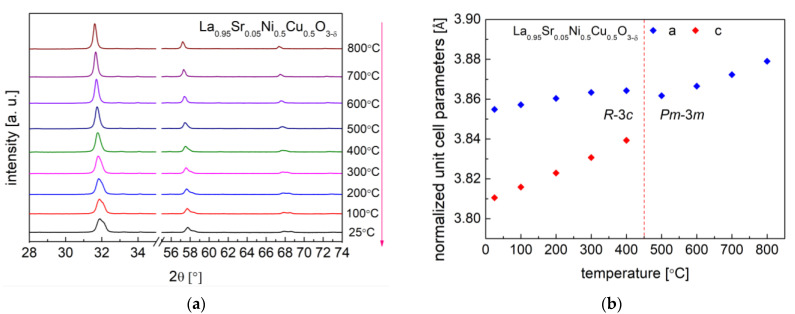
(**a**) HT-XRD diffractograms recorded for La_0.95_Sr_0.05_Ni_0.5_Cu_0.5_O_3−δ_; (**b**) Normalized unit cell parameters dependence on temperature for La_0.95_Sr_0.05_Ni_0.5_Cu_0.5_O_3−δ_.

The high-temperature XRD studies conducted between 25 °C and 800 °C in air (data recorded during cooling) presented ongoing crystal structural changes in the studied samples (Figure 4 and Figure 5). All investigated materials at high temperatures presented a regular simple perovskite structure with the *Pm*-3*m* space group. The continuous phase transition from *R*-3*c* (*a*^−^ *a*^−^ *a*^−^) to *Pm*-3*m* (*a*^0^ *a*^0^ *a*^0^) in materials was characterized by the second order. The phase transition from *R*-3*c* to the *Pm*-3*m* regular one was related to the fact that the rotation angle of the BO_6_ octahedra continually decreases with the temperature (during heating) until it reaches zero.

A similar phase transition behavior was recorded for the LaNi_0.75_Cu_0.25_O_3−δ_ and LaNi_0.5_Cu_0.5_O_3−δ_ samples in our previous work [40]. For the series of La_1−x_Sr_x_Ni_0.75_Cu_0.25_O_3−δ_ (x = 0.05 and 0.1) materials, the phase transition temperature was recorded at 550 °C and 450 °C, respectively, as shown in Figure 4. A similar situation is present for the La_0.95_Sr_0.05_Ni_0.5_Cu_0.5_O_3−δ_ oxide in Figure 5, and the phase transition occurred between 400 °C and 500 °C. As shown in Table 2, the increase in strontium content in the investigated samples decreased the phase transition temperature. It was also reported that, in the LaCrO_3_ system, the substitution of La with Sr also lowers the phase transition temperature (between *Pbnm* orthorhombic and *R*-3*c* rhombohedral structures) [53,54]. Interestingly, the La_0.95_Sr_0.05_Ni_0.5_Cu_0.5_O_3−δ_ perovskite had the lowest phase transition temperature (450 °C) among all the studied materials, while LaNi_0.75_Cu_0.25_O_3−δ_ showed the highest phase transition temperature (850 °C). The phase transition of all the studied materials did not proceed monotonously, as evidenced by the behavior of the normalized unit cell *c* parameter, which is strongly related to the evolution of oxygen content recorded in the following TG measurements.

### 3.2. Thermal Expansion Properties and Oxygen Content

**Figure 6:**
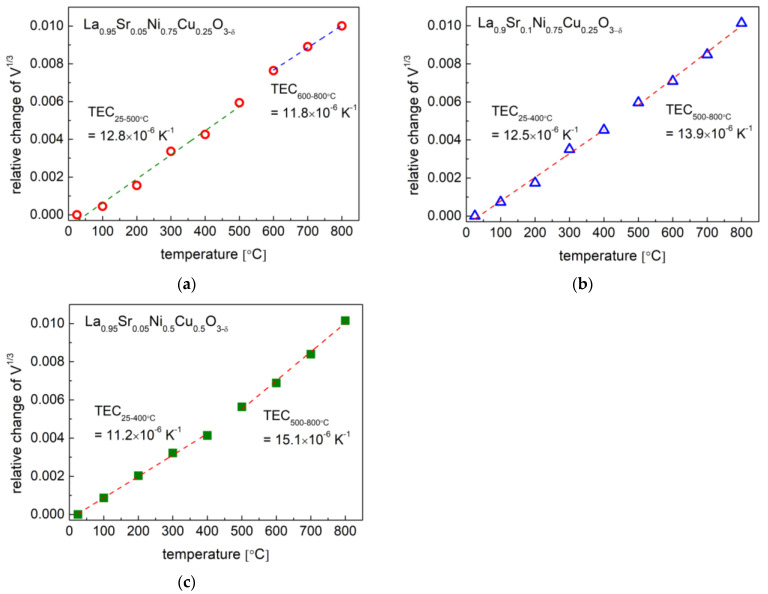
Thermal expansion coefficients calculated from HT-XRD data (V^1/3^) for (**a**) La_0.95_Sr_0.05_Ni_0.75_Cu_0.25_O_3−δ_; (**b**) La_0.9_Sr_0.1_Ni_0.75_Cu_0.25_O_3−δ_; (**c**) La_0.95_Sr_0.05_Ni_0.5_Cu_0.5_O_3−δ_.

**Figure 7:**
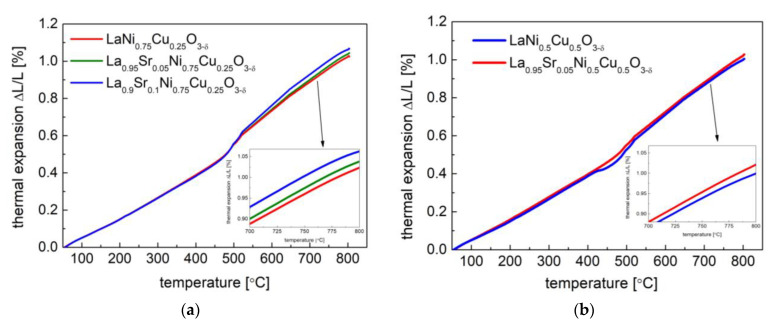
Thermal expansion behavior of (**a**) La_1−x_Sr_x_Ni_0.75_Cu_0.25_O_3−δ_ (x = 0, 0.05 and 0.1) samples; (**b**) La_1−x_Sr_x_Ni_0.5_Cu_0.5_O_3−δ_ (x = 0 and 0.05) sinters by dilatometry measurements.

The above-presented data collected from the HT-XRD studies also yielded the unit cell volume (V^1/3^) as a function of temperature, as shown in Figure 6. With the gained characteristics, it was possible to establish a thermal expansion coefficient based on the relative unit cell volume (V^1/3^) changes, and the TEC results are presented in Table 3. In general, for all the studied samples, two linear expansion behaviors with different TEC values were recorded, which is related to the phase transition and oxygen release from the material (chemical expansion effect). Similar characteristics were also observed in the dilatometry measurements, which are shown in Figure 7. The small differences between the TEC values obtained from the dilatometry measurements and calculated from the HT-XRD data are shown in Table 3, which could be associated with some of porosity in the sinters in the dilatometry measurements and the different kinetics of the phase transition in the sinters and powder. Generally, the increase in strontium content in materials increases average TEC values, which is advantageous. However, Sr doping positively contributes to the generation of oxygen vacancies in materials, thus favoring ionic transport (see the following studies). The main/significant thermal expansion contribution is from the high temperature range (linked with the chemical expansion).

The average TEC values calculated for all the samples in temperatures between 25 °C and 800 °C ranged from 13.9 × 10^−6^ K^−1^ to 15.1 × 10^−6^ K^−1^. The measured TEC values were moderate and comparable to Ni- and Cu-containing perovskites and perovskite-related oxides, including the following: La_1.5_Ba_1.5_Cu_3_O_7±δ_—15.5 × 10^−6^ K^−1^ [28]; LaNi_0.75_Cu_0.25_O_3−δ_—13.7 × 10^−6^ K^−1^ [40]; LaNi_0.5_Cu_0.5_O_3−δ_—14.5 × 10^−6^ K^−1^ [40]; PrNiO_3−δ_—12.7 × 10^−6^ K^−1^ [55]; Pr_2_CuO_4±δ_—13.0 × 10^−6^ K^−1^ [56]; Pr_2_Ni_0.5_Cu_0.5_O_4+δ_—12.7 × 10^−6^ K^−1^ [34]; and La_2_Ni_0.5_Cu_0.5_O_4+δ_—13.9 × 10^−6^ K^−1^ [34] or 12.8 × 10^−6^ K^−1^ [33]. The recorded TEC values for the La_1−x_Sr_x_Ni_1−y_Cu_y_O_3−δ_ oxides were also close to the TECs of commonly used electrolytes, including La_0.9_Sr_0.1_Ga_0.8_Mg_0.2_O_3−δ_—12.17 × 10^−6^ K^−1^, Zr_0.85_Y_0.15_O_2−δ_—10.8 × 10^−6^ K^−1^, and Ce_0.8_Gd_0.2_O_2−δ_—12.5 × 10^−6^ K^−1^ [57] (contrary to the co-containing samples [24,58]). Therefore, the delamination problem due to the TEC mismatch was alleviated, thus yielding a stable SOFC performance with the considered cathode materials.

**Figure 8:**
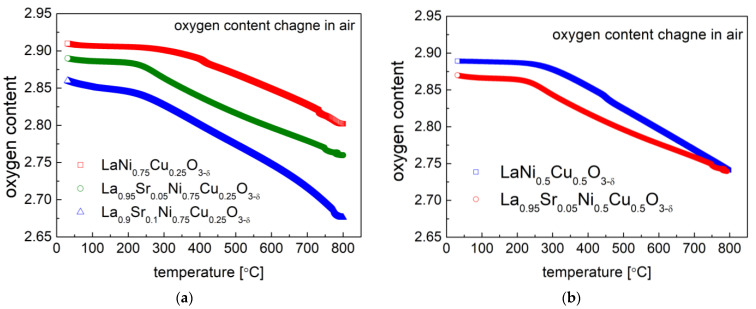
Oxygen content evolution in air for (**a**) La_1−x_Sr_x_Ni_0.75_Cu_0.25_O_3−δ_ (x = 0, 0.05 and 0.1); (**b**) La_1−x_Sr_x_Ni_0.5_Cu_0.5_O_3−δ_ (x = 0 and 0.05) oxides.

The oxygen content of the La_1−x_Sr_x_Ni_0.75_Cu_0.25_O_3−δ_ (x = 0, 0.05 and 0.1) and La_1−x_Sr_x_Ni_0.5_Cu_0.5_O_3−δ_ (x = 0 and 0.05) materials at room temperature was determined by the iodometric titration. The oxygen content change as a function of temperature is recorded in Figure 8, and the average oxidation state of B-site cations in the studied compounds at RT are presented in Table 4. In general, the increase in strontium doping at the A-site contributes to an increase in oxygen vacancies, thus decreasing the oxygen content in materials. The favorable Sr-doping effect on the formation of oxygen vacancies has also been observed in La_1−x_Sr_x_MO_3−δ_ (M = Fe, Mn) perovskites [44].

The substitution of La with Sr also led to an increase in the average oxidation state of B-site cations (Ni and Cu), causing a reduction in the unit cell volume of the studied materials (recorded in Table 1). The presence of the mixture of +3 and +2 oxidation states for Ni/Cu in La_1−x_Sr_x_Ni_1−y_Cu_y_O_3−δ_ should benefit the electronic charge transfer in materials. In the high-temperature range and in materials, additional oxygen vacancies were generated according to the following reaction:$\;O_{O}^{X}↔{1/2O}_{2}+V_{O}^{••}+{2e}^{-}$. A significant mass drop was observed for all samples above 250 °C, related to the oxygen release from the lattice. Interestingly, the La_0.9_Sr_0.1_Ni_0.75_Cu_0.25_O_3−δ_ compound exhibited the highest oxygen non-stoichiometry at RT (δ = 0.14) and 600 °C (δ = 0.25) among all the studied materials.

### 3.3. Stability and Compatibility with Solid Electrolytes

**Figure 9:**
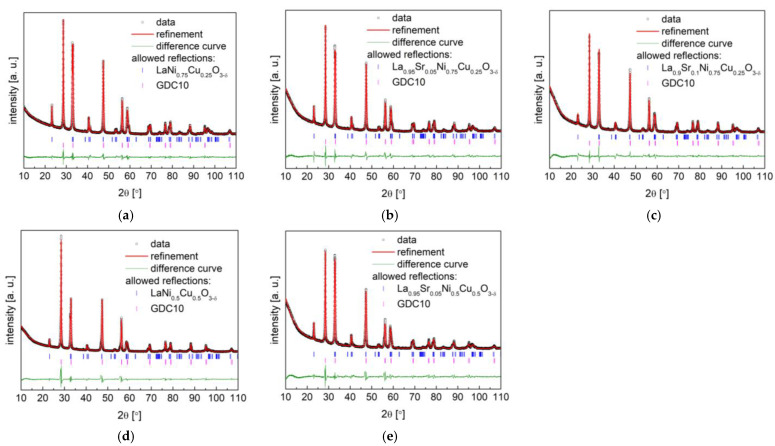
XRD diffractograms with Rietveld refinement of (**a**) LaNi_0.75_Cu_0.25_O_3−δ_; (**b**) La_0.95_Sr_0.05_Ni_0.75_Cu_0.25_O_3−δ_; (**c**) La_0.9_Sr_0.1_Ni_0.75_Cu_0.25_O_3−δ_; (**d**) LaNi_0.5_Cu_0.5_O_3−δ_; (**e**) La_0.95_Sr_0.05_Ni_0.5_Cu_0.5_O_3−δ_ with Ce_0.9_Gd_0.1_O_1.95_ electrolyte after annealing at 800 °C for 100 h.

**Figure 10:**
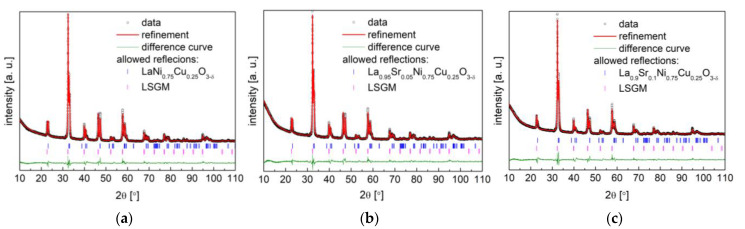

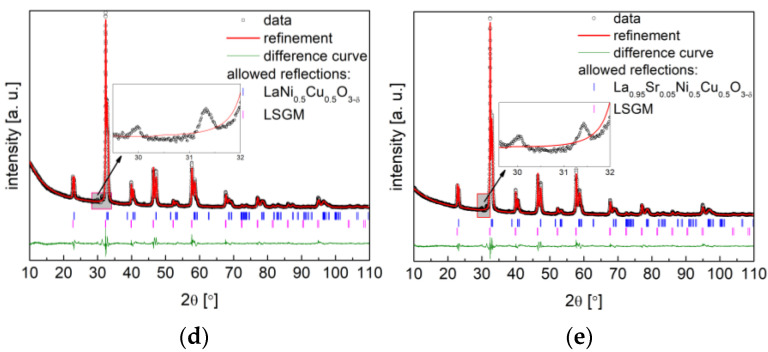
XRD diffractograms with Rietveld refinement of (**a**) LaNi_0.75_Cu_0.25_O_3−δ_; (**b**) La_0.95_Sr_0.05_Ni_0.75_Cu_0.25_O_3−δ_; (**c**) La_0.9_Sr_0.1_Ni_0.75_Cu_0.25_O_3−δ_; (**d**) LaNi_0.5_Cu_0.5_O_3−δ_; (**e**) La_0.95_Sr_0.05_Ni_0.5_Cu_0.5_O_3−δ_ with LSGM solid electrolyte after annealing in air at 800 °C for 100 h.

The chemical stability and compatibility of electrode materials with applied solid electrolytes are crucial for the stable and long-term performance of SOFCs. Long-term chemical and thermal stability studies of analyzed La_1−x_Sr_x_Ni_1−y_Cu_y_O_3−δ_ versus mostly used solid electrolytes, including CGO10, LSGM, and 8YSZ electrolytes, were conducted in air at 800 °C for 100 h. As can be observed in Figure 9, no reactivity was observed, with both the cathode materials and CGO10 phases being virtually unchanged. All studied La_1−x_Sr_x_Ni_1−y_Cu_y_O_3−δ_ cathode materials were stable and compatible with used CGO10. On the contrary, for La_1−x_Sr_x_Ni_1−y_Cu_y_O_3−δ_, some reactivity was visible towards LSGM with the emergence of additional unidentified peaks (see Figure 10), especially for the La_1−x_Sr_x_Ni_0.5_Cu_0.5_O_3−δ_ (x = 0 and 0.05) materials.

**Figure 11:**
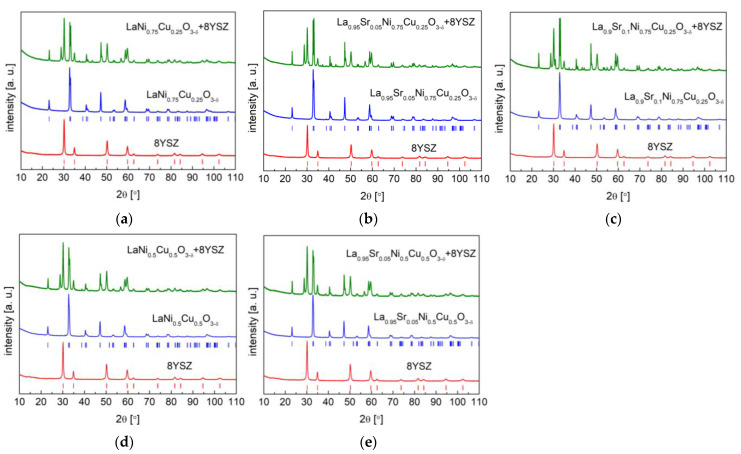
XRD diffractograms of (**a**) LaNi_0.75_Cu_0.25_O_3−δ_; (**b**) La_0.95_Sr_0.05_Ni_0.75_Cu_0.25_O_3−δ_; (**c**) La_0.9_Sr_0.1_Ni_0.75_Cu_0.25_O_3−δ_; (**d**) LaNi_0.5_Cu_0.5_O_3−δ_; (**e**) La_0.95_Sr_0.05_Ni_0.5_Cu_0.5_O_3−δ_ with 8YSZ solid electrolyte after annealing in air at 800 °C for 100 h.

Unfortunately, in the case of La_1−x_Sr_x_Ni_1−y_Cu_y_O_3−δ_ with the 8YSZ electrolyte (Figure 11), the considered cathode materials were not compatible with the studied electrolyte, presenting evident additional peaks, which limited the direct contact of the La_1−x_Sr_x_Ni_1−y_Cu_y_O_3−δ_ materials with 8YSZ in SOFCs. Therefore, for the anode-supported SOFC (Ni-8YSZ | 8YSZ | CGO10 | cathode) studied in the following section, a CGO10 buffer layer was applied to ensure a good and stable cell performance.

### 3.4. Electrochemical Performance of IT-SOFC with La_0.95_Sr_0.05_Ni_0.5_Cu_0.5_O_3−δ_

**Figure 12:**
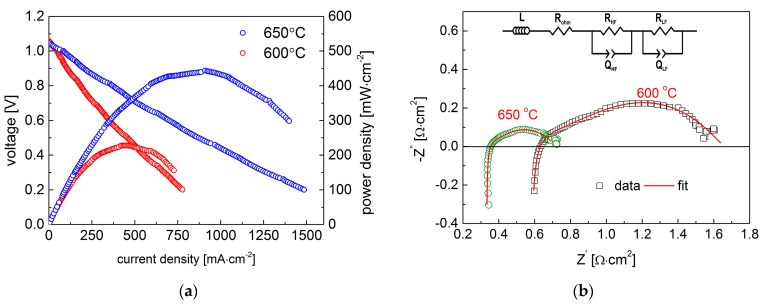
(**a**) Voltage and power density as a function of current density and (**b**) impedance spectra for anode-supported SOFC with La_0.95_Sr_0.05_Ni_0.5_Cu_0.5_O_3−δ_-based cathode.

**Figure 13:**
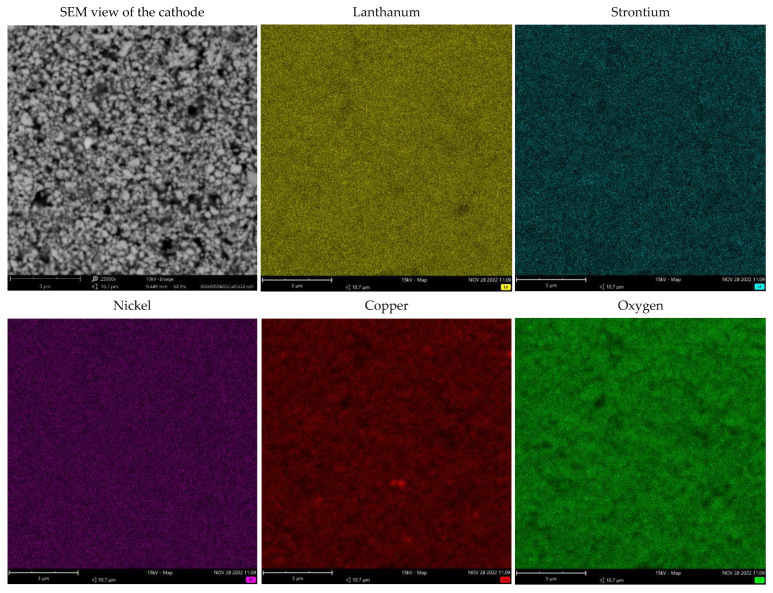
EDS map of element distribution in the La_0.95_Sr_0.05_Ni_0.5_Cu_0.5_O_3−δ_ cathode after cell tests.

A Cu-content La_0.95_Sr_0.05_Ni_0.5_Cu_0.5_O_3−δ_ oxide with low TEC (14.1 × 10^−6^ K^−1^) and high oxygen non-stoichiometry (δ = 0.22 at 600 °C) was selected as a cathode material for the IT-SOFC, working at around 600 °C (see Figure 12). The scanning electron micrograph of La_0.95_Sr_0.05_Ni_0.5_Cu_0.5_O_3−δ_ powder applied in the cathode layer is presented in Figure 13, which shows a small grain size (≤1 µm). It is worth emphasizing that the La_0.95_Sr_0.05_Ni_0.5_Cu_0.5_O_3−δ_ cathode layer was sintered at a relatively low temperature (at only 800 °C), yielding the cell fabrication process as facile and less energy-consuming, which can be related to the good sinterability of copper-containing materials and the well-attached cathode layer to CGO10 in the selected conditions.

The recorded SOFC voltage and power outputs as a function of the current density for the studied Ni-8YSZ | 8YSZ |CGO10 | La_0.95_Sr_0.05_Ni_0.5_Cu_0.5_O_3−δ_ cell are shown in Figure 12a. As can be observed, the maximum power yields reached very high values of approx. 450 mW·cm^−2^ and 230 mW·cm^−2^ in humidified hydrogen at 650 °C and 600 °C, respectively. Analyzing the shape of the voltage curves in Figure 12a, no obvious influence of activation polarization component can be observed, indicating a potential further improvement in SOFC performance. As can be seen in Table 5, the recorded power value for IT-SOFC with a La_0.95_Sr_0.05_Ni_0.5_Cu_0.5_O_3−δ_ cathode belongs to one of the best SOFC power outputs at the intermediate-temperature range, which is very encouraging. 

The EIS spectra measured for the tested IT-SOFCs are presented in Nyquist plots in Figure 12b. The measured spectra consist of two semi-arcs, in which a high frequency arc can be connected with processes taking place on the electrode and electrolyte interface (e.g., charge transfer). Additionally, a low frequency arc is associated with the electrode surface reaction, including the adsorption and dissociation of molecular oxygen [50,59]. At 600 °C, the polarization related to high frequency (R_HF_ = 0.625 Ω·cm^2^,) dominated. The values recorded for the ohmic polarization and low frequency polarization were R_ohm_ = 0.547 Ω·cm^2^ and R_LF_ = 0.491 Ω·cm^2^, respectively. Meanwhile, at 650 °C, the electrode-related polarization (R_p_ = R_HF_ + R_LF_ = 0.384 Ω·cm^2^) was comparable to ohmic polarization (R_ohm_ = 0.330 Ω·cm^2^), which indicates the possibility of further improvement in cell performance.

**Table 5:** The crystal structure, thermal expansion coefficient, compatibility with electrolytes, and SOFC performance with selected cathode materials.

Cathode Material	Crystal Structure	TEC [×10^−6^ K^−1^]	Towards Electrolyte	Cell Performance [mW·cm^−2^]	Ref.
La_0.95_Sr_0.05_Ni_0.5_Cu_0.5_O_3−δ_	*R*3-*c*	14.1	Stable with CGO10	450 at 650 °C, 230 at 600 °C in wet H_2_	This work
LaNi_0.5_Cu_0.5_O_3−δ_	*R*3-*c*	14.5	Stable with LSGM	120 at 650 °C in wet H_2_	[40]
La_1.5_Ba_1.5_Cu_3_O_7±δ_	*P*4/*mmm*	15.5	Stable with LSGM	162 at 600 °C, 250 at 650 °C in wet H_2_	[28]
LaNiO_3_	*R*-3*c*	13.7	Stable with CGO20	477 at 650 °C in wet H_2_ with LaNiO_3_/GDC composite cathode	[51,60,61]
La_0.54_Sr_0.4_6Fe_0.80_Cu_0.20_O_3−δ_	Two tetragonal phases	-	-	452 at 600 °C in H_2_	[62]
LaNi_0.6_Fe_0.4_0_3−δ_	*R*-3*c*	14.5	Stable with BZCY (below 700 °C)	431 at 650 °C, 232 at 600 °C in wet H_2_	[63,64]
La_2_Ni_0.5_Cu_0.5_O_4+δ_	*Fmmm*, *F4/mmm*	12.8, 13.9	Stable with CGO20	-	[34,56]
Nd_1.9_Ce_0.1_CuO_4_	*I*4/*mmm*	11.17	Stable with CGO	283 at 700 °C in wet H_2_	[65]
NdBa_0.5_Sr_0.5_Cu_2_O_5+δ_	*P*4/*mmm*	14.6	Stable with LSGM	343 at 750 °C	[66]
PrNiO_3−δ_	*Pnma*	12.7	Stable with CGO20	-	[55]
Pr_2_NiO_4+δ_	*Fmmm*	-	Stable with CGO25	50 at 600 °C, 80 at 650 °C in dry H_2_	[67]
Pr_2_Ni_0.5_Cu_0.5_O_4+δ_	*Bmab*	12.7	Stable with CGO20	35 at 650 °C in dry H_2_	[34]
Pr_2_CuO_4±δ_	*I*4/*mmm*	13.0	Stable with CGO20	25 at 650 °C in dry H_2_	[33]
Pr_1.7_Ca_0.3_NiO_4+δ_	*Fmmm*	-	Stable with BCGCu	96 at 650 °C, 61 at 600 °C in wet H_2_	[68]
PrBa_0.5_Sr_0.5_Cu_2_O_5+δ_	*P*4/*mmm*	14.2	Stable with LSGM	369 at 750 °C	[66]
(Pr_0.5_Nd_0.5_)_0.7_Sr_0.3_MnO_3−δ_ + SDC or 8YSZ (in molar ratio 3:2)	-	-	Stable with SDC and 8YSZ	166 at 650 °C in wet H_2_, 172 at 600 °C in wet H_2_	[69,70]
Sr_2_Fe_1.2_Mg_0.2_Mo_0.6_O_6−δ_	*Fm*-3*m*	12.9-14.6 in air; 14.6-16.7 in 5% H_2_	Stable with CGO20	-	[71]
BaCe_0.05_Fe_0.95_O_3−δ_	*Pm*-3*m*	-	Stable with SDC	315 at 650 °C, 212 at 600 °C in wet H_2_	[72]

CGO10: Ce_0.9_Gd_0.1_O_1.95_, CGO20: Ce_0.8_Gd_0.2_O_1.9_; LSGM: La_0.8_Sr_0.2_Ga_0.8_Mg_0.2_O_3−d_; CGO25: Ce_0.75_Gd_0.25_O_1.875_; SDC: Sm_0.2_Ce_0.8_O_1.95_; BCGCu: BaCe_0.89_Gd_0.1_Cu_0.01_O_3−d_; SDC: Sm_0.2_Ce_0.8_O_1.95_; BZCY: Ba(Zr_0.1_Ce_0.7_Y_0.2_)O_3−d._

In general, the cell power output (in Table 5) was strongly related to the thicknesses of the electrolytes and the types of applied electrolytes. A direct and exact comparison of power densities for different SOFCs is very difficult. Nevertheless, the power output of 450 mW cm^−2^ at 650 °C for the anode-supported SOFC with a La_0.95_Sr_0.05_Ni_0.5_Cu_0.5_O_3−δ_ cathode is still one of the best results, especially compared with reported results for anode-supported cells with a La_0.8_Sr_0.2_MnO_3_-YSZ composite cathode (261 mW cm^−2^ at 700 °C) [47], LaNiO_3_/GDC composite cathode (477 mW cm^−2^ at 650 °C) [61], (Pr_0.5_Nd_0.5_)_0.7_Sr_0.3_MnO_3−δ_–YSZ composite cathode (325 mW cm^−2^ at 700 °C) [70], and BaCe_0.05_Fe_0.95_O_3−δ_ cathode (315 mW cm^−2^ at 650 °C) [72].

The post-mortem analysis of the La_0.95_Sr_0.05_Ni_0.5_Cu_0.5_O_3−δ_ cathode was conducted after the cell performance investigation. The scanning electron micrograph of the La_0.95_Sr_0.05_Ni_0.5_Cu_0.5_O_3−δ_ cathode is shown in Figure 13. The La_0.95_Sr_0.05_Ni_0.5_Cu_0.5_O_3−δ_ cathode presented a desired porous microstructure, which was maintained after the cell measurements. Furthermore, the EDS mapping studies of element distribution presented the uniform distribution of the La, Sr, Ni, and Cu elements in the La_0.95_Sr_0.05_Ni_0.5_Cu_0.5_O_3−δ_ cathode. However, some Cu-enriched particles can be observed, which is due to the appearance of a very small amount of CuO in the synthesis.

The presented excellent electrochemical performance of fabricated anode-supported IT-SOFCs clearly shows the strontium doping in Cu-content La_1−x_Sr_x_Ni_1−y_Cu_y_O_3−δ_ perovskite oxides is a very effective strategy for the development of high-performance anode-supported SOFCs working at intermediate-temperature range.

## 4. Conclusions

Single-phase La_1−x_Sr_x_Ni_0.75_Cu_0.25_O_3−δ_ (x = 0, 0.05 and 0.1) and La_1−x_Sr_x_Ni_0.5_Cu_0.5_O_3−δ_ (x = 0 and 0.05) perovskites with strontium doping at the A-site have been successfully obtained using soft chemistry. The room-temperature crystal structure of all obtained La_1−x_Sr_x_Ni_1−y_Cu_y_O_3−δ_ compounds can be classified into the *R*-3*c* trigonal system, and phase transitions from the *R*-3*c* space group to a *Pm*-3*m* simple perovskite have been recorded at a high-temperature range by HT-XRD studies. The substitution of La with Sr in the investigated materials decreased the phase transition temperature, and La_0.95_Sr_0.05_Ni_0.5_Cu_0.5_O_3−δ_ oxide presented the lowest phase transition temperature (450 °C) among all the considered materials. Strontium doping at the A-site significantly increased the oxygen non-stoichiometry and contributed to an increase in TEC values. The thermal expansion of the studied samples was found to be anisotropic, and the obtained TEC values are similar to the most commonly applied solid electrolytes (e.g., 14.1 × 10^−6^ K^−1^ for La_0.95_Sr_0.05_Ni_0.5_Cu_0.5_O_3−δ_).

All the investigated compounds are stable and chemically compatible with GDC-10 and have some reactivity with LSGM, while they are incompatible with the 8YSZ electrolyte. The selected La_0.95_Sr_0.05_Ni_0.5_Cu_0.5_O_3−δ_ perovskite was applied to fabricate full anode-supported IT-SOFCs, and a very good power yield was documented at 445 mW·cm^−2^ and 650 °C in humidified H_2_. The results indicate that studied perovskites with a strontium doping strategy can qualify as high-performance cathode materials for anode-supported SOFCs, yielding promising cell performance in the intermediate-temperature range (around 600 °C).

## Figures and Tables

**Table 1:** Rietveld refinement results for as-synthesized La_1−x_Sr_x_Ni_1−y_Cu_y_O_3−δ_ oxides.

Composition	Space Group	a = b [Å]	c [Å]	V [Å^3^]	Density [g/cm^3^]	R_p_ [%]	R_wp_ [%]
LaNi_0.75_Cu_0.25_O_3−δ_	*R*-3*c*	5.4687(1)	13.1877(1)	341.56(1)	7.20	4.22	6.64
La_0.95_Sr_0.05_Ni_0.75_Cu_0.25_O_3−δ_	*R*-3*c*	5.4591(1)	13.1814(1)	340.21(1)	7.15	3.64	2.51
La_0.9_Sr_0.1_Ni_0.75_Cu_0.25_O_3−δ_	*R*-3*c*	5.4539(1)	13.2055(1)	340.17(1)	7.08	4.80	3.21
LaNi_0.5_Cu_0.5_O_3−δ_	*R*-3*c*	5.4730(1)	13.2166(1)	342.85(1)	7.19	4.19	2.97
La_0.95_Sr_0.05_Ni_0.5_Cu_0.5_O_3−δ_	*R*-3*c*	5.4660(1)	13.2318(1)	342.37(1)	7.14	4.24	3.00

**Table 2:** Phase transition temperature of La_1−x_Sr_x_Ni_1−y_Cu_y_O_3−δ_ oxides determined from high-temperature XRD studies in air.

Sample	Phase Transition Temperature
LaNi_0.75_Cu_0.25_O_3−δ_	850 °C [40]
La_0.95_Sr_0.05_Ni_0.75_Cu_0.25_O_3−δ_	550 °C
La_0.9_Sr_0.1_Ni_0.75_Cu_0.25_O_3−δ_	450 °C
LaNi_0.5_Cu_0.5_O_3−δ_	750 °C [40]
La_0.95_Sr_0.05_Ni_0.5_Cu_0.5_O_3−δ_	450 °C

**Table 3:** Thermal expansion coefficients [10^−6^ K^−1^] of La_1-x_Sr_x_Ni_1-y_Cu_y_O_3-δ_ samples from dilatometry studies and high-temperature XRD measurements in air.

	HT-XRD (25-400/500 °C)	HT-XRD (500–800 °C)	Dilatometry (25–400 °C)	Dilatometry (550–800 °C)	HT-XRD (25–800 °C)	Dilatometry (25–800 °C)
LaNi_0.75_Cu_0.25_O_3−δ_	-	-	11.1	15.0	-	14.3
La_0.95_Sr_0.05_Ni_0.75_Cu_0.25_O_3−δ_	12.8	11.8	11.1	15.4	12.7	14.6
La_0.9_Sr_0.1_Ni_0.75_Cu_0.25_O_3−δ_	12.5	13.9	11.1	15.8	13.2	15.1
LaNi_0.5_Cu_0.5_O_3−δ_	-	-	11.1	15.0	-	13.9
La_0.95_Sr_0.05_Ni_0.5_Cu_0.5_O_3−δ_	11.2	15.1	11.5	15.2	12.9	14.1

**Table 4:** Oxygen content and average oxidation state of B-site cations in studied compounds.

	Average Oxidation State of B-Site Cations Cu/Ni at RT	Oxygen Content at RT	Oxygen Content at 600 °C
LaNi_0.75_Cu_0.25_O_3−δ_	2.82	2.91	2.85
La_0.95_Sr_0.05_Ni_0.75_Cu_0.25_O_3−δ_	2.83	2.89	2.80
La_0.9_Sr_0.1_Ni_0.75_Cu_0.25_O_3−δ_	2.83	2.86	2.75
LaNi_0.5_Cu_0.5_O_3−δ_	2.78	2.89	2.80
La_0.95_Sr_0.05_Ni_0.5_Cu_0.5_O_3−δ_	2.79	2.87	2.78

## Data Availability

The data presented in this study are available on request from the corresponding author.
